# Mutation of the *Traj18* gene segment using TALENs to generate Natural Killer T cell deficient mice

**DOI:** 10.1038/srep27375

**Published:** 2016-06-03

**Authors:** Jingjing Zhang, Romain Bedel, S. Harsha Krovi, Kathryn D. Tuttle, Bicheng Zhang, James Gross, Laurent Gapin, Jennifer L. Matsuda

**Affiliations:** 1 Department of Immunology and Microbiology, University of Colorado Denver School of Medicine and National Jewish Health, Aurora, CO 80206, USA; 2 Department of Biomedical Research, National Jewish Health, Denver, CO 80206, USA

## Abstract

Invariant Natural Killer T (iNKT) cells are a unique subset of T lymphocytes that have been implicated in both promoting and suppressing a multitude of immune responses. In mice, iNKT cells express T cell antigen receptors (TCRs) comprising a unique TCRα rearrangement between the Trav11 and Traj18 gene segments. When paired with certain Trbv TCRβ chains, these TCRs recognize lipid antigens presented by the major histocompatibility complex (MHC) class I-like molecule, CD1d. Until recently, the sole model of iNKT deficiency targeted the Jα18, which is absolutely required to form the TCR with the appropriate antigenic specificity. However, these mice were demonstrated to have a large reduction in TCR repertoire diversity, which could confound results arising from studies using these mice. Here, we have created a new NKT-deficient mouse strain using transcription activator-like effector nuclease (TALEN) technology to only disrupt the expression of Jα18, leaving the remaining Jα repertoire unperturbed. We confirm that these mice lack iNKT cells and do not respond to lipid antigen stimulation while the development of conventional T cells, regulatory T cells, and type Ib NKT cells is normal. This new mouse strain will serve as a new model of iNKT cell deficiency to facilitate our understanding of iNKT biology.

Although the majority of αβ TCR^+^ T cells recognize peptide antigens presented by conventional polymorphic MHC I or MHC II molecules, a fraction of T cells deviates from this rule. Instead, these αβ T cells recognize lipid or glycolipid antigens presented by members of the monomorphic molecules of the CD1 family, or they recognize microbial riboflavin precursor derivatives presented by the monomorphic MR1 molecules^1^. Of these, the most extensively studied lipid-reactive T cells are the natural killer T (NKT) cells, which detect a number of glycolipid antigens in association with CD1d.

Two broad classes of NKT cells have been defined on the basis of TCR expression and antigen reactivity^2^. Most studies of these cells focus on type I, or iNKT cells (for invariant NKT cells), which are the most prevalent NKT cells in mice^3^. iNKT cells express a TCR that is the product of a canonical rearrangement between the *Trav11* (Vα14) gene segment (*Trav10* or Vα24 in human) and the *Traj18* (Jα18) gene segment, with a CDR3α region invariant at the amino acid level^4^,^5^. This Vα14 invariant chain is co-expressed with a limited set of Vβ chains, predominantly *Trbv13-2* (Vβ8.2), *Trbv29* (Vβ7) and *Trbv1* (Vβ2) in mice and *Trbv25-1* (Vβ11) in humans^4^,^5^,^6^,^7^. iNKT cells expressing these TCRs recognize several microbe-derived glycosphingolipid^8^,^9^ and diacylglycerol antigens^10^, including the prototypical glycosphingolipid antigen α-galactosylceramide (αGC)^11^,^12^, and can be identified using CD1d tetramers loaded with this antigen^13^,^14^. Because of their unique ability to rapidly and potently secrete cytokines and influence downstream responses^15^, iNKT serve as an important link between the innate and adaptive immune systems and are often regarded as potential therapeutic targets. Studies have linked iNKT cell defects with increased susceptibility to a wide range of disease processes including autoimmunity^16^, cancer^17^, and even obesity^18^. Additionally, the potential for therapies involving iNKT cells is especially attractive since the use of αGC to activate iNKT cells has proven safe in humans^19^,^20^,^21^. Ever emerging roles for this unconventional subset of lymphocytes make the study of their development and regulation both relevant and significant.

In addition to iNKT cells, there exists other CD1d-reactive T cells, which do not express the invariant Vα14-Jα18 TCR. These cells were first described when Cardell and colleagues examined the TCR usage of T cell hybridomas generated using CD4^+^ T cells isolated from MHC II-deficient mice^22^. A subset of these cells were CD1d-reactive, but instead of expressing the canonical Vα14-Jα18 TCR, the vast majority surprisingly expressed a heterogeneous TCR repertoire^22^. These CD1d-reactive cells have been named type II NKT cells or vNKT (for variant NKT cells^23^) because they do not express the canonical Vα14-Jα18 TCR, do not react with αGC, and tend to express a variable TCR repertoire^2^.

In order to study the functions of NKT cells directly *in vivo*, various genetically engineered strains of mice that lack NKT cells have been produced. Two approaches have been taken. In the first one, the Jα18 gene segment was deleted from the mouse genome through homologous gene recombination^11^. Because the amino acids coded by the Jα18 segments in the final TCRα chain of the iNKT TCR are absolutely essential to the recognition of CD1d^24^, no iNKT cells develop in absence of Jα18, as no other Jα gene segment appears to be able to compensate^11^. However, because most type II NKT cells do not use the Jα18 gene segment as part of their TCRs, this population is expected to still be intact in Jα18 deficient mice.

The second approach aimed at disrupting the expression of CD1d molecules, which is required for NKT cell development^25^,^26^,^27^. However, in contrast to the Jα18 null mouse, the CD1d deficient mice would be expected to lack both iNKT cells and type II NKT cells. Therefore, by comparing the phenotypes obtained among Jα18 deficient, CD1d deficient, and wildtype (WT) mice in various immunological models, one should be able to identify potential roles of either iNKT or type II NKT cells. Such a “trick” has now been used in many studies (see ref. 28 for review) and due to the absence of markers that distinguish type II NKT cells, this technique remains the primary means to study the potential function and influence of type II NKT cells on the immune system.

Recently, we reported that T cells derived from the original Jα18 null mouse (Tcrα-J^tm1Tgi^, called Jα18(neo) here) model of iNKT cell deficiency failed to express TCRs using *Traj* gene segments that are located 5′ of the *Traj18* gene in the genome^29^. Since non-productive rearrangements were similarly affected in these mice, we speculated that this may be due to a genetic event, possibly due to adverse consequences that the PGK-neomycin selection cassette used to generate this line might have had on transcription. The replacement of the Jα18 gene with PGK-neomycin in the reverse orientation with respect to the Jα region transcription could introduce a constitutively open chromatin configuration while the PGK promoter could serve as competition for transcription factors^30^. As a consequence, we estimated that TCRα diversity in these mice was reduced by about 60%, raising the possibility that some of the observations previously generated with these mice might be due to an incomplete TCR repertoire rather than the absence of iNKT cells.

Here, we report the generation of a new *Traj18* deficient mouse using transcription activator-like effector nuclease (TALEN) technology. Our new mouse model eliminates production and expression of the requisite iNKT cell Vα14-Jα18 junction upon recombination but preserves Jα gene usage for all other *Traj* genes to allow for proper T cell development. This new mouse provides a model to unambiguously understand the contributions of iNKT cells in *in vivo* models.

## Results

### TALEN design and *Traj18* mutant mouse generation

**Figure 1:**
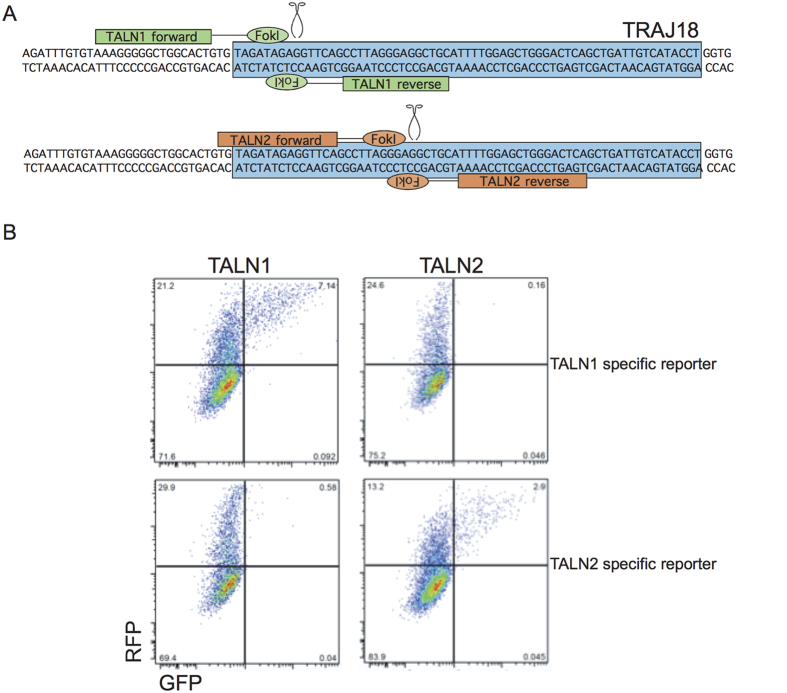
Summary of TALEN design and test of TALEN function. (**A**) Schematic of positions of forward and reverse TALENs and the DNA sequences they target. The blue box indicates the coding region of *Traj18*. (**B**) Flow cytometry of HEK293 cells after transfection with the *Traj18*-targeting TALENs and the reporter plasmids.

Two TALENs (called TALN 1 and TALN 2) were designed to target the *Traj18* gene segment (Fig. 1A). The TALENs were able to recognize and cleave the *Traj18* target sequence using an *in vitro* system, as described previously^31^. Briefly, a reporter plasmid that constitutively encodes RFP followed by the target sequence (*Traj18*) and out of frame GFP was constructed. When nuclease activity occurs, the double stranded break is repaired via error prone non-homologous end joining, creating genomic insertions or deletions (indels). Many of these indels result in frameshift mutations, putting the GFP in frame, and thus permitting its functional expression. This method allows for rapid assessment of TALEN directed mutation activity. We transfected HEK 293 cells with plasmids encoding either TALN 1 or TALN 2 and the reporter encoding the target sequences for either TALN 1 or TALN 2. Irrelevant target DNA was not cleaved, as shown by absence of GFP expression when TALN1 was co-transfected with the surrogate reporter for TALN2 and vice versa, while both TALN 1 and TALN 2 were able to cleave their targeted sequence (Fig. 1B).

**Figure 2:**
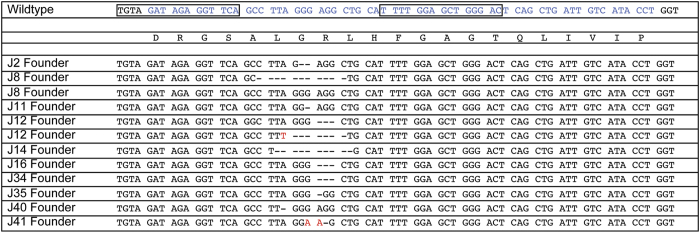
*Traj18* Sequences of founder mice. Summary of the genotype of each founder. The *Traj18* coding region is indicated in blue text with the translated amino acid sequence below. Boxes indicate the sequence targeted by the TALENs. Mutations in the founders were detected by PCR direct sequencing. Dashed lines indicate deletions while red letters indicate insertions.

RNA for each of the TALEN pair were microinjected into pure C57BL/6 zygotes. Pups derived from the injected zygotes were genotyped by direct sequencing of genomic DNA. All mice showed modifications of the *Traj18* gene sequence (not shown). Because only one *Traj18* allele might have been affected and/or both alleles might have been modified differently, we cloned PCR products amplifying the *Traj18* gene and sequenced plasmids individually. The results are summarized in Fig. 2. We selected one founder (J14) with a 10 base pair (bp) deletion in the region of the gene contributing to the CDR3. We then established the line that we refer to as Jα18(-10) here by crossing founder J14 with C57BL/6 females. Heterozygous offspring from this cross were then bred together to generate the homozygous Jα18(-10) line. The segment deleted includes the germline-encoded Jα18 CDR3 residues that are identical among mouse, rat, and human, and critical for TCR recognition and binding of the CD1d-αGC complex. Finally, because TALENs have the potential for cleaving the DNA at off-target sites, we also amplified and sequenced the top 3 potential off-target sites. All sequences were identical to the wild type genome (data not shown).

### The *Traj* repertoire diversity is preserved in Jα18(-10) mice

**Figure 3:**
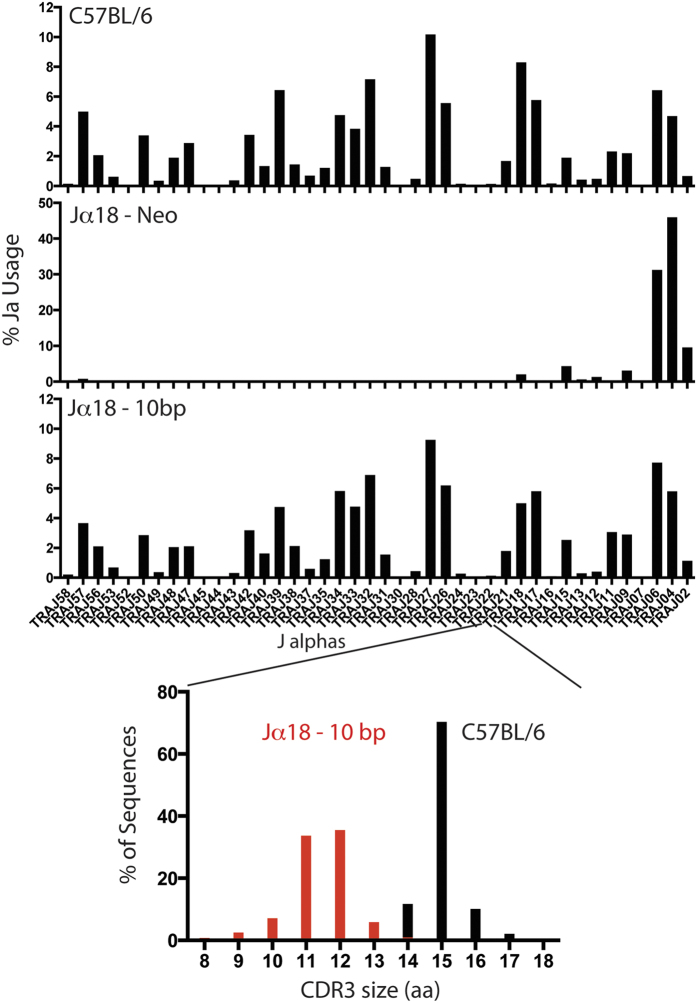
PCR analysis of the frequency of use of genes encoding Jα for productive, in-frame, rearrangements involving gene segments of the *Trav11* family in sorted CD69^−^ double-positive (CD4^+^CD8^+^) thymocytes from C57BL/6, Jα18(-10), and Jα18(neo) mice. Order of gene segments along horizontal axes (left to right) is similar to their 5′ to 3′ organization in the mouse genome. Rearrangements for were amplified by PCR with a V-specific primer and a C-specific reverse primer (above plots), followed by high-throughput sequencing with the Ion Torrent platform. Sequence analysis was performed with in-house software, and gene identity was assigned on the basis of sequence alignment with published sequences (International ImMunoGeneTics Information System).

Given the unexpected effect on the TCRα repertoire in the original Jα18(neo) mice, we wanted to examine the TCRα diversity in the Jα18(-10) mice. Pre-selection double positive thymocytes (DPs; CD4^+^CD8^+^CD69^−^) from the thymi of C57BL/6, Jα18(neo), and Jα18(-10) were sorted using flow cytometry. After generation of cDNA from these cells, we amplified Vα14 (*Trav11*) TCR rearrangements through the use of a specific forward primer for Vα14 and a reverse primer specific for the gene encoding the TCRα-chain constant region (Cα; *Trac*). PCR products were subjected to Next-generation sequencing using the Ion Torrent platform. Focusing on productive in-frame rearrangements, we found that the frequency usage of Jα gene segments in Jα18(-10) preselection DPs was comparable to C57BL/6 while the Jα18(neo) lacked TCR transcripts using Jα segments upstream of Jα18, confirming our previous data^14^ (Fig. 3).

Jα18 continues to be used in the Jα18(-10) mice, albeit at a lower percentage than in C57BL/6 WT mice (Fig. 3). Since the Jα18(-10) mouse has an intact recombination signal sequence (RSS) for the Jα18 gene, the partial gene can still undergo recombination and presumably generate productive rearrangements. In the C57BL/6 preselected repertoire, the CDR3α length of Vα14-Jα18 rearrangements is distributed between 14 and 17 amino acids, with the majority of CDR3α transcripts consisting of 15 amino acids, the length of the canonical NKT CDR3α (CVVGDRGSALGRLHF). However, in the Jα18(-10) mice, the CDR3 length distribution of Vα14-Jα18 rearrangements spanned between 9 and 13 residues. This variation was not due to a differential activity of the enzyme terminal deoxynucleotidyl transferase (TdT), which adds non-template nucleotides, but was due to the targeted 10bp deletion in Jα18 (data not shown). This equates a three to four amino acid loss in the TCRα product, represented by the shift in CDR3α size (Fig. 3).

### Vα14-Jα18 iNKT cells are absent in the Jα18(-10) mice but thymic development of conventional αβ T cells is normal

**Figure 4:**
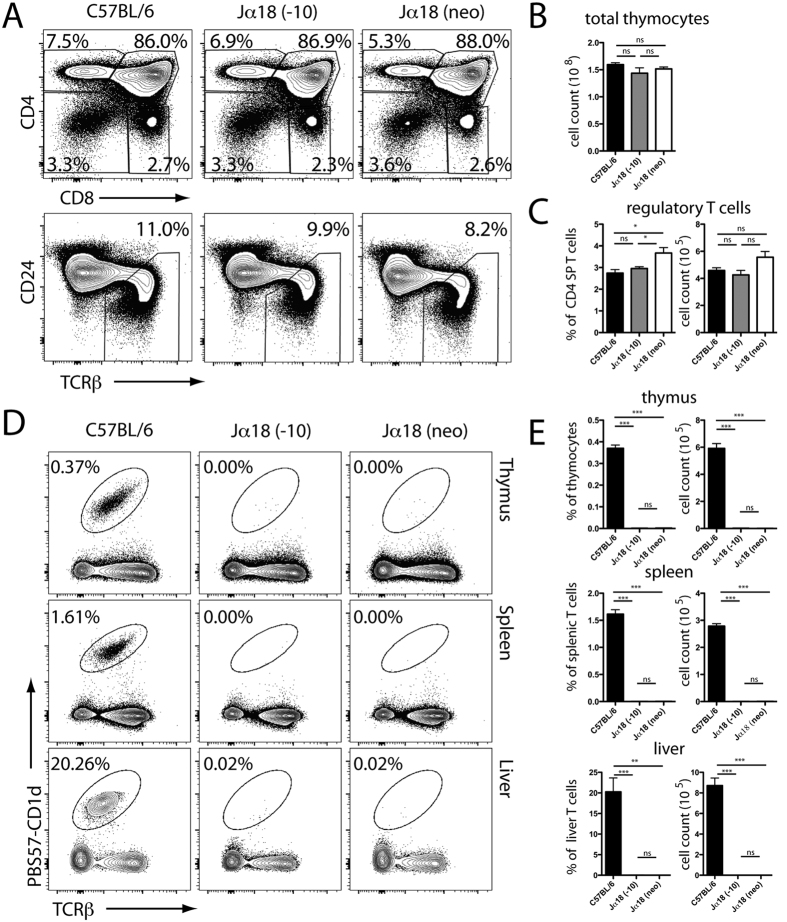
Jα18(-10) KO mice lack iNKT cells but display normal conventional T cell development. (**A**) Thymocytes from 6–8 week old C57BL/6, Jα18(-10), and Jα18(neo) were stained for indicated markers to characterize specific stages of T-cell development (data representative of *n *≥ 3). (**B**) Total thymic numbers (**C**) regulatory T cell frequencies (left) and regulatory T cell numbers (right) are summarized. Data shown represent mean ± SEM for each strain (minimum of 3 mice per strain). (**D**) Cells from thymus, spleen, and liver of 6–8 week old C57BL/6, Jα18(-10), and Jα18(neo) were stained with CD1d tetramer loaded with the α-GC analog PBS57 (PBS57-CD1d) and TCRβ to assess iNKT cells by flow cytometry (data representative of *n *≥ 3). Percentage indicated is out of all T cells in indicated organ and representative of the mean of each strain (minimum of 3 mice per strain). (**E**) Summary of the data shown in (**D**) With percentage (left) and number (right) of iNKT cells in thymus, spleen, and liver of 6–8 week old C57BL/6, Jα18(-10), and Jα18(neo) mice. Data shown represent mean ± SEM for each strain (minimum of 3 mice per strain). *P < 0.05; **P < 0.01;***P < 0.001; ns, not significant.

To assess if the deletion of 10 bp in Jα18 would affect the development of T cells, we characterized the various thymic subsets using flow cytometry. The proportions of double positive, double negative, CD4, and CD8 single positive thymocytes were identical between C57BL/6 and Jα18(-10) mice. We assessed the percentage of mature T cells, as measured by the upregulation of TCR and the downregulation of CD24 (HSA), and observed no differences (Fig. 4A). Additionally, total thymic lymphocyte cellularity was similar between C57BL/6 and Jα18(-10) mice (Fig. 4B).

We wondered if thymic regulatory cell (Treg) development, which has more stringent requirements for TCR specificity and self reactivity than bulk conventional αβ thymocytes (reviewed in^32^) was perturbed. Assessment by percentage and number revealed that Treg development was not affected by the 10 bp Jα18 deletion (Fig. 4C). Surprisingly, the Jα18(neo) had a greater percentage of regulatory T cells in the CD4 pool, which could be a byproduct of Jα repertoire skewing (Fig. 4C).

Next, we assessed the proportion and iNKT cell numbers in the thymus, spleen, and liver by flow cytometry using PBS57 (an αGC analog) –loaded CD1d tetramers that specifically stain iNKT cells^33^. Over one million events were collected per sample in all organs to ensure that the data generated were representative. As seen in Fig. 3D, the proportion and numbers of iNKT cells in the thymus, spleen, or liver of the Jα18(-10) mice were close to background levels, similar to what is found with the original Jα18 (neo) mice (Fig. 4D,E).

### Type Ib NKT cells in the Jα18(-10) mice

**Figure 5:**
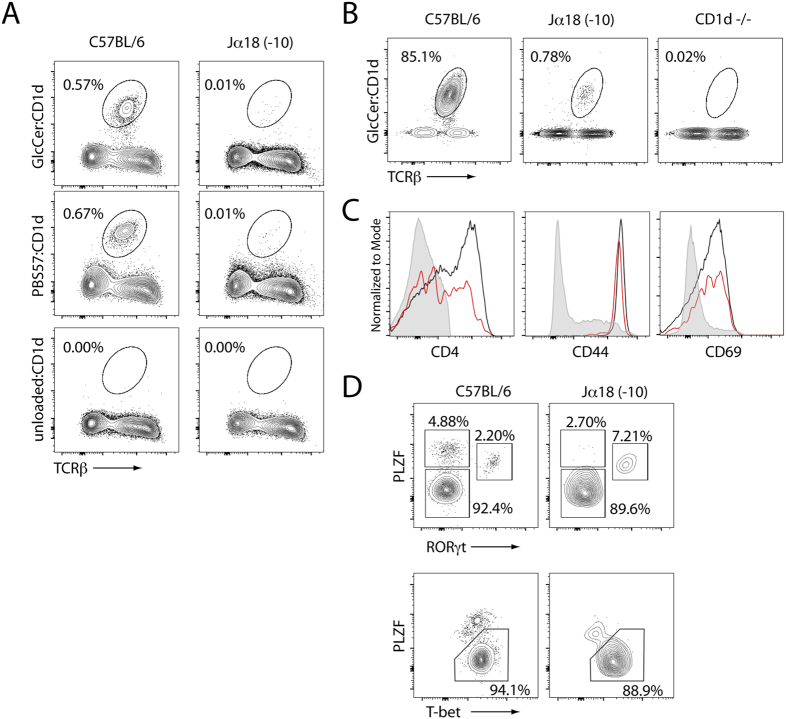
Type Ib NKT cells in the Jα18(-10) thymus. (**A**) Thymocytes from 6–8 week old C57BL/6 and Jα18(-10) were stained with CD1d tetramer loaded with the α-GC analog PBS57 (PBS57-CD1d) or α-GlcCer and analyzed by flow cytometry (data representative of *n* ≥ 3). (**B**) α-GlcCer/CD1d tetramer reactive thymocytes were enriched by MACS beads from C57BL/6 and pooled (3–5 mice per strain) Jα18(-10) and CD1d-/- thymi. Enriched cells were stained with TCRβ and α-GlcCer/CD1d tetramer and assessed by flow cytometry. (**C**) Expression of CD4, CD44, and CD69 in enriched populations from C57BL/6 (black) and Jα18(-10) (red) thymi were compared to the staining control population (DN, TCRβ^−^; gray filled). Dead cells were excluded from analysis using a viability dye. (**D**) Enriched cells from (**A**) were stained with intracellular PLZF, T-bet and RORγt to identify NKT1, NKT2 and NKT17 subsets. Dead cells were excluded from analysis.

In addition to iNKT cells that use the canonical Vα14-Jα18 rearrangement, another minor population of αGC-reactive NKT cells was recently described^34^. These cells express a canonical Vα10-Jα50 TCRα chain and were named type Ib NKT cells^34^. They were originally identified in the Jα18(neo) mice, which minimally express TCRα rearrangements that involve the Jα50 segment (see Fig. 3 and^29^). Because the new Jα18(-10) mice had restored normal *Traj* frequency distribution, we wondered if we would detect a larger Vα10Jα50 type Ib NKT population in these mice. Although type Ib NKT cells recognize α-GC/CD1d complexes, they show a preference for the recognition of α-glucosylceramide (α-GlcCer) presented by CD1d^34^. Therefore, we stained the thymocytes from C57BL/6 and Jα18(-10) mice with both of these tetramers. A minor population, representing about 0.01% of total thymocytes could be detected using both tetramers but was absent from CD1d-deficient mice (Fig. 5A). To ensure the legitimacy of this population, we enriched the cells using α-GlcCer/CD1d tetramers and magnetic bead cell isolation (Fig. 5B). Using this enrichment, we could detect a small but genuine population of type Ib NKT cells in the thymus of Jα18(-10) mice. The cells included CD4^+^ and CD4^−^ subsets, were CD44^high^ and CD69^int^ (Fig. 5C), as previously reported^34^. We also examined the expression of the three major transcription factors (PLZF, T-bet and RORγt) that were recently used to define functionally distinct iNKT cell subsets^35^. NKT1 cells are defined as T-bet^hi^, PLZF^lo^ RORγt^lo^, NKT2 cells are T-bet^lo^, PLZF^hi^ and RORγt^lo^, while NKT17 cells are T-bet^lo^, PLZF^int^ and RORγt^hi^. As seen in Fig. 4D, the proportion of NKT2 cells was lower and the proportion of NKT17 cells was higher in NKT type Ib as compared to Vα14Jα18 type I iNKT cells (Fig. 5D). Altogether, these results confirm a population of Jα18 independent NKT cells that are neither classical type I nor type II and are probably not limited in frequency solely due to a reduced Jα repertoire.

### Jα18(-10) mice do not respond to αGC challenge *in vivo*

**Figure 6:**
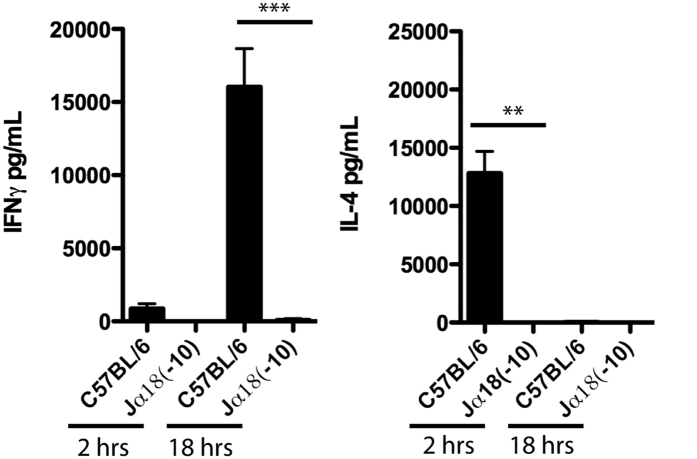
Jα18(-10) mice do not respond to αGC challenge *in vivo.* C57BL/6 and Jα18(-10) mice received 2 μg of αGC intravenously. Serum was collected at 2 and 18 hours after injection. IL-4 and IFN-γ was quantified by enzyme-linked immunosorbent assay. Data shown represent mean ± SEM for each group (minimum of 3 mice per group). **P < 0.01; ***P < 0.001.

Upon stimulation with αGC, iNKT cells are selectively activated and rapidly secrete large quantities of IL-4 and IFN-γ cytokines^14^,^36^. As seen in Fig. 5, upon αGC injection into C57BL/6 mice, large quantities of IL-4 and some IFN-γ were detected in the serum two hours post-injection. At 18 hours post-injection, large quantities of IFN-γ, mostly due to the transactivation of NK cells by activated iNKT cells^36^,^37^, were detected in the serum of C57BL/6 mice. In contrast, we did not detect any of these cytokines when the experiment was performed with Jα18(-10) mice (Fig. 6). It is likely that the cytokine quantities released by activated type Ib NKT cells were too low to be captured by our assay. Altogether, these results further demonstrate the absence of functionality associated with the iNKT cell population in the Jα18(-10) mice.

## Discussion

We have previously reported that the original Jα18 null mouse had defects in the TCRα repertoire beyond the absence of the Jα18 gene segment^29^. As a consequence, we estimated that a large portion of TCR diversity was lost in these animals. Since the creation of the Jα18(neo) in 1997, these mice have been the sole model of type I NKT cell deficiency. To avoid the confounding and unintended results due to reduced TCR diversity, every study in the twenty-year span that used these mice as a model of NKT cell deficiency or to differentiate between type I versus type II NKT cell consequences would benefit from a reassessment. We have created a mouse that is deficient only for type I NKT cells that use the invariant Vα14-Jα18 TCR chain. This new Jα18 null mouse has considerable advantages over the original line, preserving the Jα diversity of the WT animal while eliminating expression of the invariant NKT TCR. Therefore, we expect not only the full restoration of T cell repertoire diversity but also the restoration of other unique T cell populations with limited and nearly exclusive *Traj* usage that might have been lost in the Jα18(neo) mice. For example, TCRs expressed by mucosal-associated invariant T (MAIT) cells are composed of rearrangements involving *Trav1* and primarily *Traj33* in mice^38^. Because *Traj33* is located 5′ of *Traj18* in the *Traj* locus, it is likely that the overall population size of MAIT cells is affected in the Jα18(neo) mice. The restoration of Jαs proximal to Jα18 presumably allows for normal development of this population in the Jα18(-10) mice. Interestingly, frequencies of Jα18 independent type Ib NKT cells were not appreciably increased despite more *Traj50* representation in the preselected repertoire. Thus, other factors must govern the number of developing type Ib NKT cells such as limited thymic niche, ligand availability or genetics since these cells are more abundant in the BALB/c than C57BL/6 background^34^.

Recently, Chandra *et al.* generated and described another Jα18 deficient using bacterial artificial chromosome and cre/lox technology to remove both *Traj18* and the neomycin cassette^39^. We, instead, elected to use targeted genome engineering and created our line with TALEN technology. Using this method, we prevented expression of the iNKT TCR without eliminating Jα18 expression. In the Jα18(-10) mice, the preselected T cells using the Jα18 gene segment had CDR3α lengths on average 3–4 amino acids shorter than what is found in wild-type mice. Presumably, some of these TCRs can be positively selected and expressed. Additionally, the Jα18 RSS is undisturbed, allowing for normal recombination events. Nonetheless, by disrupting the size of the CDR3α loops that can be generated, we completely abrogated the development of iNKT cells, in agreement with the importance that this loop has in interacting with the antigen-CD1d complex^40^.

We have shown that the Jα18(-10) mouse is a precise model of iNKT deficiency while maintaining the development of other CD1d-restricted T cells, which do not require the Vα14-Jα18 rearrangement. Unlike the Jα18(neo) mice that have reduced TCR repertoire diversity, the TCR repertoire in Jα18(-10) mice appears similar to WT mice. We believe this novel mouse will serve as a new standard model for iNKT cell deficiency and eliminate the confounding factors that have surfaced with the original mouse model.

## Methods

### Mice

The Jα18(-10) mouse was produced by the Mouse Genetic Core facility at NJH. The CD1d-/- mice and Jα18(neo) mice have been described previously^11^,^26^. C57BL/6 were purchased from Jackson Laboratories. All mice were used between 6 to 12 weeks and were age matched for each experiment. All mice were raised in a specific pathogen-free environment at the Biology Resource Center in National Jewish Health or the University of Colorado Anschutz Medical Campus Vivarium. All Experiments were approved by the NJH and the University of Colorado Institutional Animal Care and Use Committees and performed in accordance with their guidelines.

### Lymphocyte Isolation

Single cell suspensions were prepared from the thymus, spleen, and liver by manual disruption using syringe plunger. The liver was perfused with PBS then cut into small pieces, disrupted with a syringe plunger, and liver lymphocytes were isolated by centrifugation using a 33% (vol/vol) Percoll gradient (GE Healthcare).

### High-Throughput Sequencing

CD69^−^CD4^+^CD8^+^ pre-selection double positive thymocytes were sorted by flow cytometry and washed twice in ice-cold PBS. Total RNA was extracted using the RNeasy Mini Kit (Qiagen). cDNA was made using SuperScript III Reverse Transcriptase (Invitrogen). The Vα14-Cα region was amplified using specific Vα14 (5′-TACAGTGTGACCCCCGACAAC-3′) and Cα (5′-GAGGGTGCTGTCCTGAGACCGAG-3′) primers with required specific Ion Torrent tags. Purified PCR products were sent for high-throughput sequencing using the Ion Torrent platform. Sequence analysis was done with in-house software, and gene identity was assigned on the basis of sequence alignment with published sequences (International ImMunoGeneTics Information System).

### Flow Cytometry

CD1d-PBS57 was obtained from the National Institutes of Health Tetramer Core Facility. CD1d-α-glucosylceramide tetramers were generously provided by the Dr. Dale Godfrey lab. The complete list of surface antibodies used is as follows: From BD Biosciences: anti-TCRβ (H57-597), anti-CD69 (H1.2F3), anti-CD122 (TM-β1), anti-CD8α (53-6.7), anti-CD45.2 (104); From BioLegend: CD44 (IM7); From eBioscience: anti-CD25 (PC61.5), anti-B220 (RA3-6B2), anti-MHCII (I-A/I-E) (M5/114.15.2), anti-CD4 (RM4-5), anti-CD24 (M1/69). Surface antibody staining was done then cells were fixed and permeabilized using the FoxP3 buffer set (eBioscience). Fixed and permeabilized cells were incubated with intracellular antibodies including anti-PLZF (Mags.21F7; eBioscience), anti-T-bet (4B10; BioLegend), anti-RORγt (Q31-378; BD Biosciences), or anti-Foxp3 (MF23; BD Biosciences).

Cells were analyzed on a BD LSRFortessa (BD Biosciences) and data were processed with FlowJo software (TreeStar).

### Enrichment of CD1d reactive thymocytes

Thymocytes were enriched for CD1d-α-galactosylceramide reactive cells by incubating thymocyte cell suspensions with PE conjugated CD1d-α-glucosylceramide for 45 minutes at 4 °C, then incubated with anti-PE magnetic microbeads (Miltenyi Biotec) for 15 minutes at 4 °C, followed by separation by using an autoMACS Pro Separator (Miltenyi Biotec) according to manufacturer’s instructions. Cells were then later surface stained, fix/pearmeabalized, and then intracellular stained for flow cytometry. Fixable Viability Dye from Affymetrix eBioscience was used to exclude dead cells in enrichment experiments.

### *In vivo* Cytokine Response Quantification

Mice were administered vehicle or 2 μg of αGC (Alexis Biochemicals) by intravenous injection. Blood was collected at 2 hours and 18 hours post injection and serum was isolated using Z-gel microtubes (Sarstedt). Serum cytokine levels were measured by ELISA using Mouse Th1/Th2 ELISA Ready-SET-Go! (Affymetrix eBioscience) according to manufacturer’s instructions.

### Statistical analysis

Prism software (GraphPad) was used for statistical analysis. Unpaired, two-tailed Student’s t tests were used for generation of p-values.

## Additional Information

**How to cite this article**: Zhang, J. *et al.* Mutation of the *Traj18* gene segment using TALENs to generate Natural Killer T cell deficient mice. *Sci. Rep.* **6**, 27375; doi: 10.1038/srep27375 (2016).
